# Ultrasonographic and radiographic features of a true gastrogastric intussusception with spontaneous resolution in a cat

**DOI:** 10.1002/vms3.920

**Published:** 2022-08-25

**Authors:** Elodie E. Huguet, Elisa Spoldi, Aria Guarino, Kirsten L. Cooke, John F. Roberts

**Affiliations:** ^1^ Department of Small Animal Clinical Sciences College of Veterinary Medicine University of Florida Gainesville Florida USA; ^2^ Department of Comparative Diagnostic & Population Medicine College of Veterinary Medicine University of Florida Gainesville Florida USA

**Keywords:** feline, stomach, vomiting, ultrasonography, radiography

## Abstract

An 11‐year‐old, female spayed, domestic shorthair cat with a 1‐week history of vomiting was diagnosed with a gastrogastric intussusception using ultrasound. Distinguishing ultrasonographic findings included invagination of the gastric fundus into the body and were correlated to radiographs acquired at the time of the evaluation. Spontaneous resolution of the gastrogastric intussusception was observed on a positive‐contrast upper gastrointestinal fluoroscopic study done the following day. Due to worsening comorbidities, which most significantly included chronic renal disease and pancreatitis, and declining quality of life, the patient was humanely euthanized 10 months later. Necropsy revealed no gross and histopathologic abnormalities associated with the stomach. A definitive cause for the intussusception remains unknown.

## Graphical abstract

This report represents the first description of a true gastrogastric intussusception in a feline patient and highlights the utility of ultrasound and radiographs to achieve a diagnosis. While the clinical significance remained unknown, true gastrogastric intussusceptions in feline patients may be self‐limited.

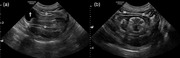

## 1 INTRODUCTION

As previously defined, in contrast to pylorogastric or duodenogastric intussusceptions, true gastrogastric intussusceptions represent invagination of the gastric fundus into the lumen of the gastric body (Graham et al., 2020; Vikram et al., 2006). This finding has been rarely reported in human literature and is predominantly diagnosed in older human patients (greater than 65 years of age), while only young dogs are affected in the very few available peer‐reviewed veterinary publications (Behrooz & Cleasby, 2018; Graham et al., 2020; Testault et al., 2022; Vikram et al., 2006). The current report describes a similar finding in a mature cat, suggesting that a different age distribution may exist between species. A report by Testault et al. (2022) evaluated intussusceptions in cats, regardless of their classification, and found a bimodal age distribution, which also differs from reports of young dogs being more commonly affected. Feline intussusceptions are prevalently enteroenteric, more often reported to be jejunojejunal and ileocolic, and rarely involve the stomach (Burkitt et al., 2009; Levitt & Bauer, 1992). In the absence of identifiable cause, intussusceptions in young cats are more commonly considered idiopathic, while neoplasia is a more prevalent reported aetiology in older cats (Burkitt et al., 2009; Levitt & Bauer, 1992). Comorbidities are not uncommon but diverse with an undetermined predisposing role in the formation of intussusceptions in cats (Burkitt et al., 2009). Cats most often present with signs of lethargy and anorexia of a variable duration, ranging between 1 day to 1 month (Burkitt et al., 2009; Levitt & Bauer, 1992; Patsikas et al., 2003). Vomiting is seen less commonly with an inconsistent incidence reported in the literature (Burkitt et al., 2009; Patsikas et al., 2003). A higher frequency of intussusceptions is described in Maine Coons (Haider et al., 2019). A predisposing cause in this population of cats remains unknown (Haider et al., 2019; Verschoof et al., 2015). Intussusceptions involving the stomach are rarely reported in cats and are primarily gastroesophageal intussusceptions in a few case reports (Martinez et al., 2001; Tayler et al., 2021). In dogs, gastroesophageal, gastrogastric and gastroduodenal intussusceptions are described. True gastrogastric intussusceptions are the less frequently reported type. A distinct aetiology remains unknown; although a most recent report describes a benign polyp and gastritis as a possible cause. A similar disease has not been previously described in cat, making this case report the first description of a gastrogastric intussusception in a feline patient.

## 2 CASE HISTORY

An 11‐year‐old, female spayed, domestic shorthair cat presented to the Small Animal Internal Medicine Service at the University of Florida's College of Veterinary Medicine for a recheck evaluation following an approximately 10‐day long history of vomiting with a normal appetite. Prior to presentation, the cat had been managed the last 1.5 years for multiple comorbidities, including acromegaly treated with stereotactic radiotherapy, intermittently‐controlled diabetes mellitus, pancreatic pseudocysts/cysts and nodules, chronic diet‐responsible small bowel diarrhoea, International Renal Interest Society stage 2 chronic kidney disease, and non‐regenerative anaemia. The newly developed vomiting of either fluid or undigested kibble was associated with abdominal contractions and would occur once daily shortly after eating dry kibble (Feline Multifunction Renal Support + Hydrolyzed Protein dry food, Royal Canin, S.A., Aimargues, France). The vomiting resolved 4 days prior to presentation after the diet was switched to a canned formulation (Feline Selected Protein PR Canned Cat Food, Royal Canin, S.A., Aimargues, France). A few self‐limited and conservatively managed episodes of vomiting per months were historically reported. The cat received 11 units of insulin Lantus (Insulin Glargine injection 100 units/ml, Sanofi, S.A., Paris, France) subcutaneously every 12 h with one/third cup of dry kibble or third/fourth of food. At the time of presentation, readings from a previously placed FreeStyle Libre Flash Glucose Monitoring System (Abbott Diabetes Care Inc., Alameda, CA, USA) showed an average interstitial glucose of 114 mg/dl with occasional non‐clinical hypoglycaemia. The hypoglycaemia would occur occasionally, and only after the patient had vomited. It was suspected that the vomiting precipitated the hypoglycaemia due to decreased nutrient assimilation.

On presentation, the cat was bright, alert, and responsive with normal vital parameters. Physical examination revealed a new 2/6 left apical systolic heart murmur, static acromegaly associated facial features, and static moderate epaxial muscle wasting, with a body condition score of 5/9 (ideal). No organomegaly or signs of discomfort were noted on abdominal palpation. A complete blood count revealed a mild leucocytosis (16.1 × 10^3^ while blood cells/µl; reference interval 5.4–15.4 white blood cells/µl), mild hyperfibrinogenaemia (0.4 g/dl; reference interval 0.1–0.3 g/dl), and neutrophilia (11.0 × 10^3^ neutrophils/µl; reference interval 2.3–9.8 neutrophils/µl). The cat had a non‐regenerative anaemia with a packed cell volume of 16% (reference interval 30%–45%) and an aggregate reticulocyte count of 0 reticulocytes/µl (reference interval 0–30,000/µl). A serum biochemistry panel revealed an elevated BUN (74 mg/dl, reference interval 19–35 mg/dl) and creatinine (2.45 mg/dl, reference interval 0.8–1.8 mg/dl) with mild hyponatremia (147.7 mg/dl, reference interval 148–155.3 mg/dl) and hyperphosphatemia (5.8 mg/dl, reference interval 3.0–5.7 mg/dl). Blood glucose measurement was elevated (261 mg/dl, reference interval 72.1–156.1 mg/dl). Additional serum biochemistry findings included elevated creatinine kinase (1550 IU/L, reference interval 20–440 IU/L) and aspartate aminotransferase (54 IU/L, reference interval 13–45 IU/L) levels. The cat was fasted for 12 h prior to the appointment and had received no insulin that morning.

**FIGURE 1:**
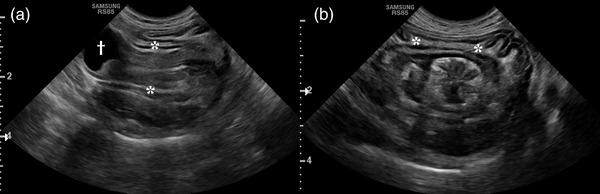
Longitudinal (a) and transverse (b) B‐mode ultrasonographic images of the gastrogastric intussusception. Concentric superimposition of the gastric wall layers of the intussusceptum (fundus and orad part of the gastric body) and intussuscipiens (gastric body) were noted. A small amount of hyperechoic fat was identified within this region of involution (asterisks). The orad portion of the cardia contained a small amount of anechoic fluid (obelus)

The cat underwent a complete abdominal ultrasound as part of the recheck evaluation. The images were acquired using a combination of microconvex and linear transducers (CF4‐9 MHz, LA4‐18B MHz and L3‐12A MHz; RS85, Samsung Healthcare, Danvers, MA, USA). The ultrasonographic evaluation identified involution of an approximately 4 cm long portion of the stomach, including primarily the gastric fundus and orad aspect of the gastric body into the aborad aspect of the gastric body in a normograde direction, which represented a gastrogastric intussusception and resulted in concentric superimposition of the wall layers of the intussuscipiens (outer segment) and intussusceptum (inner segment) (Figure 1a,b). A small amount of hyperechoic fat and vessels, distinguished by the presence of blood flow on colour and power Doppler interrogation, were identified between the intussuscipiens and intussusceptum. The visible orad portion of the cardia contained a small amount of anechoic fluid. The pylorus and duodenum were within normal limits.

Other ultrasonographic findings correlated to an abdominal ultrasound done approximately 9 months prior included a progressively enlarged and now heterogeneously hypoechoic lobular pancreas with a new right pancreatic mass (1.7 × 1.5 cm in maximal dimension), progressive hypoechoic nodules and cyst‐like lesions, and new moderate to severe regional steatitis. A primary differential diagnosis of acute on chronic pancreatitis with diffuse pancreatic cysts/pseudocysts and nodular hyperplasia was considered primarily. For the right‐sided pancreatic mass, considered aetiologies included primary pancreatic neoplasia (e.g. carcinoma), chronic abscess, nodular hyperplasia, or less likely metastatic neoplasia. The liver and spleen remained mildly enlarged, likely associated with acromegaly, as well as vacuolar hepatopathy in correlation with the diabetes mellitus and extramedullary haematopoiesis in response to the chronic anaemia.

**FIGURE 2:**
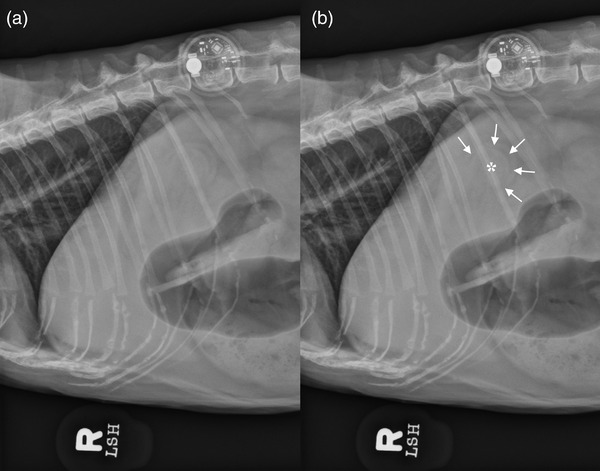
Right lateral radiographic projection of the abdomen with (a) and without (b) annotations. A focal soft tissue opaque bulge (asterisk) is identified between the region of the gastric body and fundus and has smooth and rounded margins outlined by a small amount of intraluminal gas with a crescent‐shape distribution (arrows). Superimposed with the right craniodorsal abdominal wall, there is a metal opaque electronic device, representing a FreeStyle Libre blood glucose sensor

Three‐view abdominal radiographs, including left lateral, right lateral, and ventrodorsal projections, were acquired for teaching purposes (lateral projections 95kVp 2.5mAs; ventrodorsal projection 100 kVp 5mAs, Canon CxDI 55G, Canon Medical Systems, Otawara, Japan). A focal soft tissue opaque bulge was identified in the region between the gastric body and fundus and was outlined by a small amount of intraluminal gas with a crescent‐shape distribution (Figure 2a,b), compatible with the ultrasonographic findings of gastrogastric intussusception. The sole identification of gas within the pyloric region on ultrasound supported the presence of atypically distributed gas within a dorsally displaced pyloric antrum on radiographs. No gastric or oesophageal dilation was identified to support the presence of mechanical obstruction.

**FIGURE 3:**
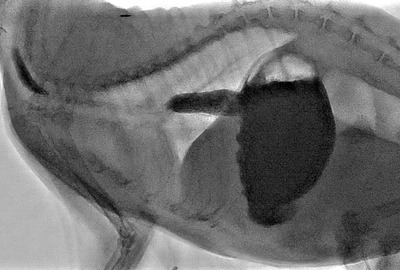
Videofluoroscopic image highlighting normal passage of food bolus through the lower gastroesophageal sphincter and normal contrast filling of the gastric lumen, suggesting spontaneous resolution of the previously diagnosed gastrogastric intussusception

The following day, a positive contrast upper gastrointestinal study was performed under fluoroscopic guidance for evaluation of gastric transit (68 kVp, 10 mA, 15 fps, Phillips Healthcare, Amsterdam, the Netherlands). The patient was imaged in a standing position, and fluoroscopic video loops were obtained during administration of barium sulphate‐coated moist cat food for a total volume of approximately 260 ml. Normal passage of barium‐coated food boluses was observed through the lower gastroesophageal sphincter and cardia, and followed by progressive filling of the gastric lumen. At this time, no abnormalities associated with the gastric wall and lumen were identified, suggesting spontaneous resolution of the previously diagnosed gastrogastric intussusception (Figure 3). The cat was discharged and medically managed for her comorbidities for an additional 10 months before she was humanely euthanized due to progression of the comorbidities. A few weeks prior to humane euthanasia, a computed tomography (CT) of the chest, abdomen, and pelvis was performed due to palpable progressive enlargement of the pancreas and confirmed progressive enlargement of the pancreatic lesions. The CT did not reveal an obvious gastric pathology, and no recurrence of the gastrogastric intussusception. Following humane euthanasia, a necropsy was performed. The stomach was grossly and histopathologically normal. The most significant findings included the presence of severe, neutrophilic and histiocytic to necrotizing chronic pancreatitis with fibrosis, nodular hyperplasia and exocrine cysts. As identified on ultrasound, the pancreas contained a large mass, which was compatible with an exocrine pancreatic carcinoma on histopathology. Additionally, hypertrophic cardiomyopathy with concurrent myxomatous mitral valve disease was identified as a cause for the heart murmur. The systemic vasculature had mild to moderate smooth muscle hyperplasia and perivascular fibrosis suggestive of chronic hypertension.

## 3 DISCUSSION

To the authors’ knowledge, this report represents the first documented case of a true gastrogastric intussusception in a cat. The clinical signs of the cat in the current report were acute, lasting a few days, and differentiated from the shorter duration (<24 h) of the acute clinical signs of vomiting and hematemesis described in dogs and did not include the clinical signs of abdominal pain and weight loss described in human patients (Behrooz & Cleasby, 2018; Graham et al., 2020; Vikram et al., 2006). Additionally, the reported dogs had no prior history of vomiting, unlike the cat in this report, and acute relapse of the intermittent mild vomiting episodes was also considered (Graham et al., 2020; Testault et al., 2022). Chronic intermittent clinical signs of vomiting in a dog are also reported in a small case series (Testault et al., 2022). The primary concern of vomiting in the cat differed from the more prevalent signs of lethargy and anorexia reported in cats with intussusception (Burkitt et al., 2009; Patsikas et al., 2003). The gastrogastric intussusception was considered self‐resolved since additional imaging failed to support persistence or recurrence of the gastrogastric intussusception. Self‐resolution of pylorogastric intussusceptions of unknown aetiology has been described in dogs (de Brito Galvao et al., 2011; Huml et al., 1992). The patient's clinical signs persisted following resolution of the intussusception and resolved following transition to a canned diet formulation alone. While it is possible that the intussusception caused the vomiting and apparent intolerance for dry kibble, it is also possible that the vomiting occurred secondary to other comorbidities such as diet‐responsive enteropathy or pancreatitis.

Radiographs and ultrasound were useful imaging modalities for the diagnosis of true gastrogastric intussusception in cats. On radiographs, atypical distribution of intraluminal gas within the stomach and the presence of a soft tissue opaque bulge in the region of the gastric body, fundus, and/or cardia may raise suspicion for a gastrogastric intussusception. Ultrasonography helped confirm the presence of, and further characterize the gastrogastric intussusception, as well as identify comorbidities. Alternatively, CT has been described as a useful imaging modality for the identification of true gastrogastric intussusception in dogs (Graham et al., 2020).

True gastrogastric intussusceptions in older human patients are most commonly associated with benign and malignant gastric tumours (Graham et al., 2020; Huml et al., 1992; Vikram et al., 2006). No gastric mass was identified in the current case to support a similar aetiology; however, a sizable pancreatic neoplastic mass was present adjacent to the pylorus. On necropsy, the patient had severe pancreatitis with nodular hyperplasia and cysts, as well as evidence of chronic portal hypertension. Exacerbation of these conditions combined with the fasted state of the patient at the time of presentation may have resulted in imperceptible gastritis, gastric wall oedema, increased intraabdominal pressure, or gastric hypermotility that precipitated the development of the intussusception. Gastritis, gastric wall hyperplasia and/or gastric wall oedema was confirmed in most reported canine case of gastrogastric intussusceptions and has previously been described as a sequela of pancreatitis in dogs (Graham et al., 2020; Murakami et al., 2019; Testault et al., 2022). While the ultrasonographic features of the current case did not support a diagnosis of gastric wall oedema, the presence of pancreatitis and evidence of chronic portal hypertension on histopathology did not rule out the possibility of imperceptible gastric wall oedema at the time of diagnosis. Gastric wall oedema was identified on histopathology in one of the canine cases; however, this finding was considered likely secondary to compromised venous and lymphatic return (Graham et al., 2020). Furthermore, portal hypertension has previously been postulated as a cause for gastrogastric intussusception in the human literature in the absence of a tumour and may be a considered cause for the gastrogastric intussusception in the reported feline patient (Behrooz & Cleasby, 2018).

The fasted state of the patient may have resulted in increased gastric migrating motor complex contractions and hypermotility. However, it is unlikely that the fasted state alone was the cause of the intussusception because patients are commonly fasted for abdominal imaging without this finding. Alternatively, the patient's severe pancreatomegaly may have resulted in increased intraabdominal pressure, predisposing to gastrogastric intussusception formation.

In conclusion, this is the first case report of a true gastrogastric intussusception in cat. Ultrasound and radiographs were useful modalities for the diagnosis of the true gastrogastric intussusception in the current case and shared imaging characteristics similar to prior veterinary reports. The gastrogastric intussusception spontaneously resolved and additional imaging failed to support recurrence. A cause for the true gastrogastric intussusception in this cat remained uncertain. The patient's clinical decline leading and ensuing euthanasia at a later time was deemed unrelated to any gastric pathology.

## CONFLICTS OF INTEREST

The authors declare no conflict of interest.

## AUTHOR CONTRIBUTIONS

*Conception and design*: Elodie E. Huguet and Elisa Spoldi. *Acquisition of data*: Elodie E. Huguet, Elisa Spoldi, Aria Guarino, Kirsten L. Cooke, and John F. Roberts. *Analysis and interpretation of data*: Elodie E. Huguet, Elisa Spoldi, Aria Guarino, Kirsten L. Cooke, and John F. Roberts. *Drafting the article*: Elodie E. Huguet. *Revising article for intellectual content*: Elodie E. Huguet, Elisa Spoldi, Aria Guarino, Kirsten L. Cooke, and John F. Roberts. *Final approval of the completed article*: Elodie E. Huguet, Elisa Spoldi, Aria Guarino, Kirsten L. Cooke, John F. Roberts.

## FUNDING INFORMATION

None.

## ETHICS STATEMENT

Approval from the Ethics Committee was not needed for the completion of this case report.

### PEER REVIEW

I would not like my name to appear with my report on Publons https://publons.com/publon/10.1002/vms3.920

## Data Availability

The data that support the findings of this study are available from the corresponding author upon reasonable request.
